# Transient rho-associated coiled-coil containing kinase (ROCK) inhibition on human retinal pigment epithelium results in persistent Rho/ROCK downregulation

**DOI:** 10.1016/j.bbrep.2020.100841

**Published:** 2020-11-18

**Authors:** Shohei Kitahata, Hinako Ichikawa, Yuji Tanaka, Tatsuya Inoue, Kazuaki Kadonosono

**Affiliations:** a Department of Ophthalmology and Micro-technology, Yokohama City University, Yokohama, Japan; b Division of Medicine, Interdisciplinary Graduate School of Medicine and Engineering, University of Yamanashi, Yamanashi, Japan

**Keywords:** Rho-associated coiled-coil containing kinase (ROCK) inhibitor, Transient exposure, Retinal pigment epithelium, Cell preservation

## Abstract

Retinal pigment epithelium (RPE) cells is the outermost layer of the retina and RPE dysfunction is a key factor in the disease pathogenesis of age-related macular degeneration (AMD). Transplantation therapy using induced pluripotent stem cell (iPSC)-derived RPEs has recently received much attention as a treatment for AMD. Preserving these cells under the best possible conditions is important, and preservation methods using Y-27632 have been reported. Rho-associated coiled-coil containing kinase (ROCK) inhibitors are known to inhibit cell death, emerging as important drug candidates for stem cell differentiation and regenerative medicine. However, it has recently been shown that ROCK inhibitors may have a vasodilatory effect on human retinal arterioles, a side effect that should ideally be avoided in RPE transplantation. Although ROCK inhibitors hold great potential, optimizing efficacy while minimizing adverse reactions is critical for translation into a clinical treatment. We examined the effect of transient exposure of RPE cells to ROCK inhibitor Y-27632 to determine whether the extracellular presence of the drug is necessary for ongoing Rho/ROCK downregulation. Human RPE cells were subcultured as a suspension for 4 h in drug-free medium following exposure to Y-27632 for 2 h. A Y-27632 concentration of >10 μM improved cell survival beyond 4 h and cell proliferation in recovery culture medium. ROCK2 expression levels were specifically downregulated by Y-27632 in the Rho/ROCK signaling pathway. In conclusion, we demonstrated that the effect of Y-27632 is not dependent on its extracellular availability and can last beyond the 2 h of exposure. The lasting Rho/ROCK signaling pathway downregulation by Y-27632 suggests that RPE cell transplantation with ROCK inhibitor-free media is possible, which can minimize side effects to host tissue and have wider implications for transplantation methods requiring ROCK inhibition.

## Highlights

• Transient exposure of retinal pigment epithelium to ROCK inhibitor Y-27632 increased cell viability and proliferation.
• Intracellular uptake of Y-27632 led to persistent downregulation of the Rho/ROCK signaling pathway.
• This effect of Y-27632 sustained even after its removal from the medium.

## 1 Introduction

Retinal pigment epithelium (RPE) cells is the outermost layer of the retina and associated with functional integrity of both photoreceptors and the choroidal vasculature. And RPE dysfunction is known to play an important role in the disease pathogenesis of age-related macular degeneration (AMD) [1]. Current treatments for AMD involving the intravitreal injection of anti-vascular endothelial growth factor (VEGF) are insufficient for complete recovery and RPE cell therapy derived from embryonic stem (ES) cells and induced pluripotent stem cells (iPSC) has been initiated [2,3]. Recently, our group found that Y-27632 meaningfully suppressed cell death in human iPSC-RPE cell suspensions [4]; this discovery has the potential to solve practical problems concerning cell preservation and transport. With respect to preservation, Rho-associated coiled-coil containing kinases (ROCK) inhibitors have attracted attention. ROCK is a downstream effector of the small GTP-binding proteins RhoA and RhoC [5] that has been implicated in various cellular processes including cell proliferation, differentiation, cytokinesis, motility, adhesion, and cytoskeletal arrangement [6]. ROCK activation is known to be closely related to apoptosis [7] with accompanying disruption of actin organization that causes significant reduction in cell shape and movement [8]. It has been reported that ROCK inhibitor Y-27632 suppresses myosin light chain phosphorylation and subsequent cell death [7]. An important use of Y-27632 has been demonstrated in improving cloning efficiency of dissociated single human ES cells through the suppression of myosin hyperactivation [8]. These antiapoptotic effects of Y-27632 are expected to be clinically applied in various fields such as regenerative medicine and ophthalmology [4,9,10].

Y-27632 was recently approved for glaucoma treatment [11], and a clinical trial for its use in corneal endothelial cell transplantation is in progress [9]. Severe retinal diseases are less regenerative and pose great challenges in treatment, and various treatments have been applied for these intractable diseases such as stem cell-derived cell transplantation therapy [3,[12], [13], [14]]. Although ROCK inhibitors are recommended for such retinal diseases, safety confirmation and risk mitigation are the first priorities and should not be ignored [15,16]. ROCK inhibition causes the vasodilation of human retinal vessels [17], potentially limiting its use in facilitating RPE cell transplantation due to involvement with functional integrity of both the retinal and choroidal vasculature. Therefore, it is necessary to develop an effective method to minimize such risks without compromising their benefits.

**Fig. 1:**
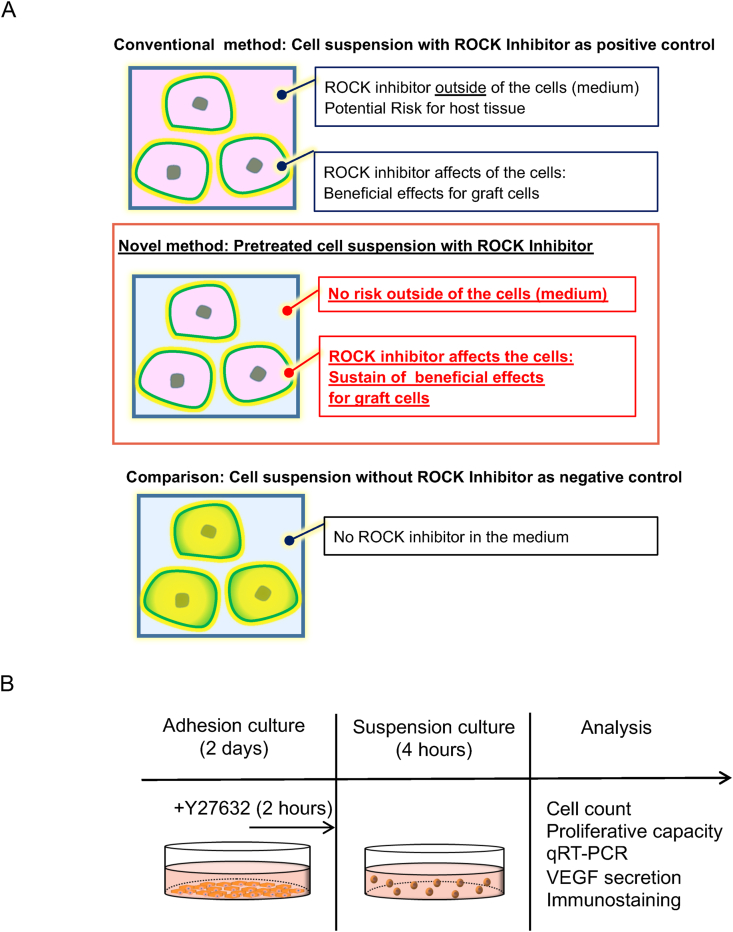
Study scheme: conceptual feature (A) and experimental workflow (B) of this research. (A) Conceptual feature of this research was to identify the difference between the conventional method (Y-27632 is contained in the medium and cells) and the proposed novel method “pretreatment” (Y-27632 is not contained in the medium but in cells). (B) Experimental workflow of this research. RPE cells were cultured for 2 days as adhesion (transient exposure was performed 2 h before its end) and then suspended under one of the three conditions (PC, NC, and pretreatment). After suspension culture, cells were utilized for each analysis in this study.

In this study, we first examined the effect of transient exposure of RPE cells to Y-27632, aiming to eliminate unnecessary adverse effects on host tissue during transplantation by removing the ROCK inhibitor from the culture medium (Fig. 1A) and to determine whether ROCK inhibition in RPE cells is sustained after transient exposure. Transient exposure of RPE to Y-27632 (Pretreatment) were comapared with treated RPE cells (positive control [PC]) and untreated RPE cells (negative control [NC]) by evaluating cell survival, proliferation, VEGF secretion and cell adhesion.

## 2 Materials and methods

### 2.1 Cell culture

Human primary RPE cells (#00194987, Lonza) were cultured in a maintenance medium (DMEM/F12 [7:3]) supplemented with B27 (Thermo Fisher Scientific) and 2 mM l-glutamine (FUJIFILM Wako Pure Chemical Corporation) and 10 ng/mL bFGF and 0.5 μM SB43152 on Laminin-511 E8 fragment (iMatrix-511, Nippi)-coated plates in a humidified incubator at 37 °C in 5% CO_2_. The medium was changed every 2–3 days. Upon reaching confluence, the cells were incubated with 0.25% trypsin-EDTA (Life Technologies) at 37 °C for 10 min. Trypsin was quickly neutralized using fetal bovine serum-containing medium. RPE cells were seeded in maintenance medium and adhesion medium (DMEM/F12 containing 10% FBS) on a new iMatrix-511-coated plate. We used the fifth passage of the human RPE cells.

### 2.2 Experimental design

RPE cells were seeded in maintenance medium and adhesion medium on iMatrix-511-coated plate for 2 days. The cells were exposed to Y-27632 (FUJIFILM Wako Pure Chemical Corporation) only for 2 h just before the trypsin treatment. The Y-27632 concentrations used were 1, 5, 10, 30, and 100 μM. RPE cells were incubated in 0.25% trypsin-EDTA at 37 °C for 10 min and quickly neutralized using fetal bovine serum containing medium. Then, the suspensions were prepared in a medium without ROCK inhibitors. Cell suspensions were prepared in maintenance medium and preserved for 4 h at 37 °C (Fig. 1B). Adherent cultures were seeded at 0.6 × 10^5^ cells/cm^2^. Suspension culture was started at 1.0 × 10^5^ cells/mL in maintenance medium. The PCs and NCs were defined as those exposed and not exposed to Y-27632, respectively.

### 2.3 Cell viability

Cell viability was assessed 4 h or 8 h after suspension culture using 0.4% trypan blue stain (Bio-Rad). Trypan blue solution (0.4%, 10 μL) was transferred to a test tube and 10 μL of the cell suspension was added and mixed; 10 μL of the mixture was then pipetted into the hemocytometer. We counted the trypan blue-negative cells (viable) and stained (dead) cells separately by hemocytometer. However, we utilized the total live cell number as the dead RPE cells were destroyed.

### 2.4 MTS assay

To evaluate cell proliferative capacity, we used CellTiter 96 Aqueous One Solution Cell Proliferation Assay (Promega), based on tetrazolium compound (3-[4,5-dimethylthiazol-2-yl]-5-[3-carboxymethoxyphenyl]-2-[4-sulfophenyl]-2H-tetrazolium; MTS). Cells were seeded in a 96-well iMatrix-511-coated plate after Y-27632 treatment or no treatment. The reagent was added to each sample each day. After a 2-h incubation at 37 °C, absorbance was measured at 492 nm in a microplate reader (ChroMate, Awareness Technology).

### 2.5 RNA isolation and quantitative RT-PCR (qRT-PCR)

Total RNA was extracted from RPE cells (n = 4 for each group) using the miRNeasy Mini Kit (Qiagen) according to the manufacturer's instructions. Each sample was added to 700 μL of QIAzol Lysis Reagent, and the contents were vortexed. Afterward, 140 μL of chloroform was added and the tube was shaken vigorously for 15 s. The tube was placed on the benchtop at room temperature for 3 min and centrifuged for 15 min at 12,000×*g* at 4 °C. The upper aqueous phase was transferred to a 2-mL collection tube, and 525 μL of 100% ethanol was added and mixed thoroughly by pipetting up and down several times. The sample, including any precipitate that might have formed, was transferred to the RNeasy Mini spin column in a 2-mL collection tube and centrifuged at 8000×*g* for 15 s at room temperature. The flow-through was discarded. Buffer RWT (700 μL) was added to the RNeasy Mini spin column and the column was centrifuged for 15 s at 8000×*g* for washing. The flow-through was discarded. Subsequently, 500 μL of buffer RPE was added to the RNeasy Mini spin column and the column was centrifuged for 15 s at 8000×*g* for washing. The flow-through was discarded. Another 500 μL of buffer RPE was added to the RNeasy Mini spin column, and the column was centrifuged for 2 min at 8000×*g* to dry the RNeasy Mini spin column membrane. Finally, the contents of the RNeasy Mini spin column were transferred to a new 1.5-mL collection tube, and 50 μL of RNase-free water was added directly to the RNeasy Mini spin column membrane; the column was then centrifuged for 1 min at 8000×*g* to elute the RNA. First-strand cDNA was synthesized from 1 μg of total RNA using the PrimeScript RT Master Mix (Takara Bio Inc.). qRT-PCR was performed with CFX96 Touch Real-Time PCR Detection System (Bio-Rad) and SsoAdvanced Universal SYBR Green Supermix (Bio-Rad). Primer sequences used were as follows: *RhoA* forward: 5′-CCATCATCCTGGTTGGGAAT -3′, reverse: 5′-CATGTACCCAAAAGCGCCA-3′, *ROCK2* forward: 5′-GCTTCAAAGGAGCCCAGATTT -3′, reverse: 5′-TCGACAGCTTGCCCCAAA -3′, *β-actin* forward: 5′- CCAACCGCGAGAAGATGA -3′, reverse: 5′- CCAGAGGCGTACAGGGATAG -3′. Target gene expression was normalized using the 2^−ΔΔCt^ method from the Ct values of the respective mRNAs relative to the housekeeping gene *β-actin*.

### 2.6 Immunocytochemistry

Human RPE cells were fixed with 4% paraformaldehyde for 20 min, permeabilized with 0.1% Triton X-100 in phosphate-buffered saline for 30 min, blocked with 1.5% goat serum (FUJIFILM Wako Pure Chemical Corporation) for 1 h at room temperature (RT), and incubated with primary antibody for 1 h at RT. The primary antibody was mouse anti-ZO-1 (ZO1-1A12, Thermo Fisher Scientific) at 1:500. Next, the cells were incubated with secondary antibodies for 1 h at RT. The secondary antibody was goat antimouse Alexa Fluor 488 (Life Technologies) at 1:1000. Nuclei were stained with 4,6-diamindino-2-phenylindole (DAPI) at 1:100 (Bio-Rad). Labeled cells were observed using a fluorescence microscope (BZ-X700, Keyence).

### 2.7 VEGF assessment

To evaluate VEGF secretion of human RPE cells, we used Human VEGF SimpleStep ELISA Kit (ab222510, abcam). After suspension culture, RPE cells were seeded at 0.6 × 10^5^ cells/well into 48-well iMatrix-511-coated plates and cultured at 37 °C for 13 days. Then replaced with fresh medium and supernatants were collected after 24 h. All media were collected from wells and kept in frozen storage until assayed. The amount of VEGF secreted per cell was calculated by dividing VEGF concentration in cell medium by the number of cells in the culture well. Data was quantified in comparison to VEGF standards.

### 2.8 Statistical analysis

The data were expressed as mean ± SED, with p < 0.05 considered as statistically significant. Statistical analysis was conducted using the SPSS statistical package (SPSS Inc. version 22). A paired *t*-test and one-way ANOVA with Tukey's post hoc pairwise comparisons were performed to compare each result.

## 3 Results

### 3.1 Transient exposure to Y-27632 increases viability of human RPE cells

**Fig. 2:**
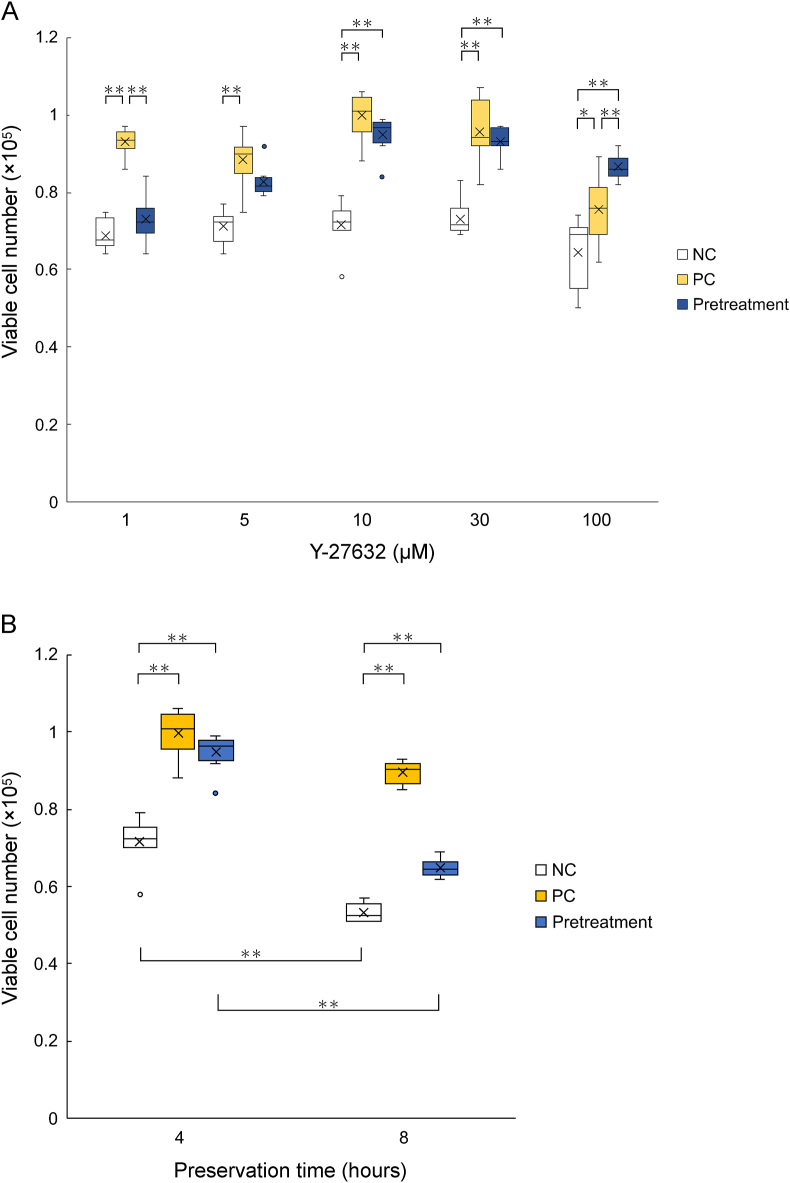
The number of viable human RPE cells after suspension culture in each condition: negative control (NC), positive control (PC), and pretreatment condition. (A) Evaluation of the number of viable cells after 4 h of suspension culture in each condition. The Y-27632 concentrations used were 1, 5, 10, 30, and 100 μM. (B) Evaluation of the number of viable cells after suspension culture with 10 μM Y-27632 for 4 and 8 h. (A, B) n = 5; mean ± SED; *p < 0.05 and **p < 0.01 compared with all other conditions; one-way ANOVA with Tukey's post hoc pairwise comparisons test.

To examine the effect of transient exposure to Y-27632 on human RPE cell suspensions, cell viability was analyzed using trypan blue staining. Initially, adherent human RPE cells were cultured with Y-27632 at various concentrations (1, 5, 10, 30, and 100 μM) for 2 h. These concentrations were selected based on the retinal migration of commercially available eye drops [19]. Subsequently, the RPE cells were trypsinized, centrifuged, and suspended in Y-27632-free media. The NC group did not receive Y-27632 treatment at either stage. The number of viable cells was significantly increased with transient exposure to Y-27632 at all concentrations (PC). Moreover, during transient exposure at concentrations 10, 30, and 100 μM, the number of viable cells also increased compared with non-exposure to Y-27632 (NC) (Fig. 2A and Table 1).

**Table 1:** Mean values ( × 10^4^) and calculated viable cell frequency (%) from samples at various ROCK inhibitor concentration and conditions. Mean ± SD is indicated. n = 5.

Condition			
ROCK inhibitor concentration (μl)	NC	PC	Pretreatment
1	6.86 ± 0.41 (68.6 ± 4.1%)	9.30 ± 0.35 (93.0 ± 3.5%)	7.29 ± 0.58 (72.9 ± 5.8%)
5	7.13 ± 0.43 (71.3 ± 4.3%)	8.84 ± 0.65 (88.4 ± 6.5%)	8.28 ± 0.41 (82.8 ± 4.1%)
10	7.15 ± 0.62 (71.5 ± 6.2%)	9.98 ± 0.60 (99.8 ± 6.0%)	9.49 ± 0.49 (94.9 ± 4.9%)
30	7.31 ± 0.47 (73.1 ± 4.7%)	9.58 ± 0.81 (95.8 ± 8.1%)	9.33 ± 0.37 (93.3 ± 3.7%)
100	6.45 ± 0.91 (64.5 ± 9.1%)	7.55 ± 0.85 (75.5 ± 8.5%)	8.65 ± 0.32 (86.5 ± 3.2%)

To confirm the duration of the effect of transient exposure to Y-27632, cell suspensions were preserved for 8 h at 37 °C. The number of viable cells decreased in 10 μM Y-27632 after 8 h in the NC group. On the other hand, treatment durations of 4 h and 8 h in the PC group produced no significant difference in cell viability (Fig. 2B). Interestingly, a significant reduction in cell viability was observed between the 4 h and 8 h pretreatment groups, indicating that the effect of transient exposure to Y-27632 on cell viability decreased over time.

### 3.2 Functional assessment of human RPE cells after transient exposure to Y-27632

**Fig. 3:**
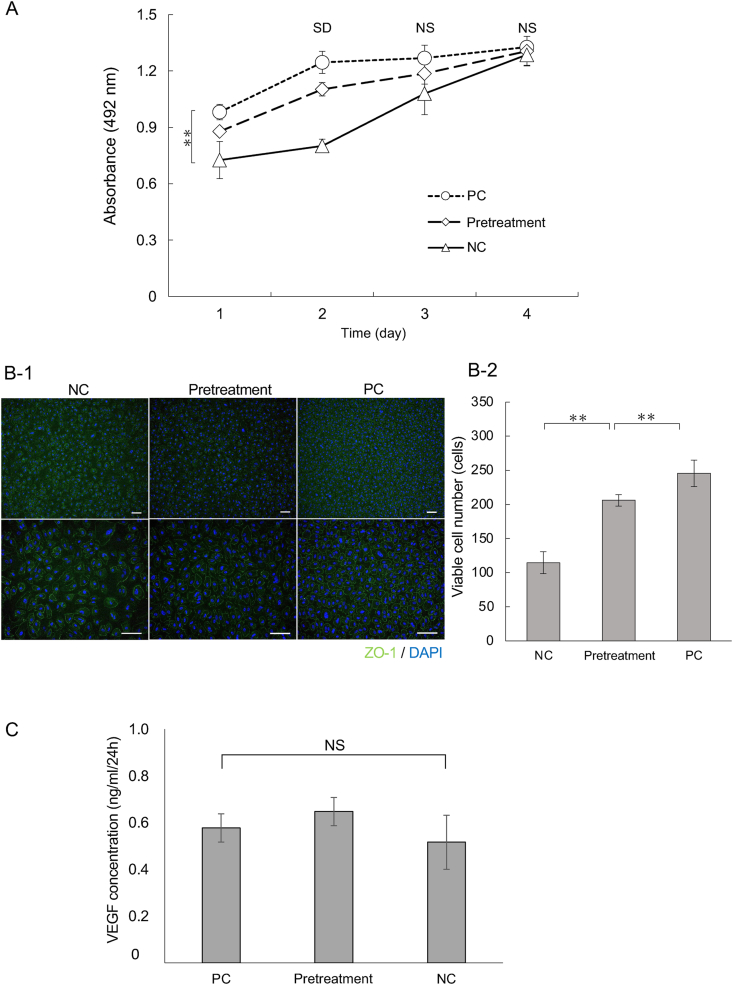
Functional assessment and cell signature in recovery culture. (A) Cell proliferation assessment by MTS assay: RPE cells after 4 h suspension culture in NC, PC, and pretreatment conditions were plated for recovery culture, and cell proliferation was subsequently evaluated by MTS assay each day from days 1–4 (n = 3). (B) Epithelial morphology and then number of viable cells at confluence during recovery culture at 10 days after suspension culture. Cells were plated into a 12-well laminin-coated plate and cultured for 10 days using RPE cells after suspension culture. (B-1) Morphology was assessed by immunofluorescence microscopy (green, ZO-1). Upper is low magnification (10 × ) and lower is high magnification (20 × ). Scale bars = 100 μm. (B-2) The number of viable cells in the image of samples prepared for (B-1) was quantified (n = 3; mean ± SED; *p < 0.05 and **p < 0.01). (C) VEGF secretion quantified using an ELISA assay in preserved RPE cells after 13 days in a recovery culture (n = 4). NC, negative control; PC, positive control. (For interpretation of the references to colour in this figure legend, the reader is referred to the Web version of this article.)

Because ROCK inhibitor treatment can affect important cell characteristics including viability and the secretion of key proteins, we recovered RPE cells for culture after 4 h of preservation in suspension to confirm the cells’ function. We utilized the MTS cell proliferation assay and ELISA to examine the secretion of VEGF protein and compared samples. To further assess the prosurvival effects of Y-27632, we recovered human RPE cell suspensions exposed to 10 μM Y-27632 and cultured them for 4 days. We used the MTS assay and compared the PC and NC. Cell proliferation checked from days 1–4 after seeding in recovered RPE cells showed significant difference at days 1 and 2. The number of viable cells was observed in the order of PC, pretreatment, and NC. Although there was a significant difference between PC and NC at day 1, each condition showed a significant difference at day 2. At days 3 and 4, human RPE cell proliferation was similar in all conditions (Fig. 3A).

Next, we assessed RPE cell morphology with phase-contrast microscopy and zonula occludens-1 (ZO-1) expression. Ten days after recovery cultures were established, ZO-1 and DAPI were examined. ZO-1 expression was stable throughout all conditions (Figs. 3B–1, 2). We also examined VEGF, which is a signal protein produced by RPE cells that stimulates the formation of blood vessels [20]. Thirteen days after the recovery cultures were established, VEGF secretion was measured; no significant difference in VEGF secretion was seen among all the experimental conditions (Fig. 3C).

### 3.3 Transient exposure to Y-27632 suppressed ROCK activation in human RPE cells

**Fig. 4:**
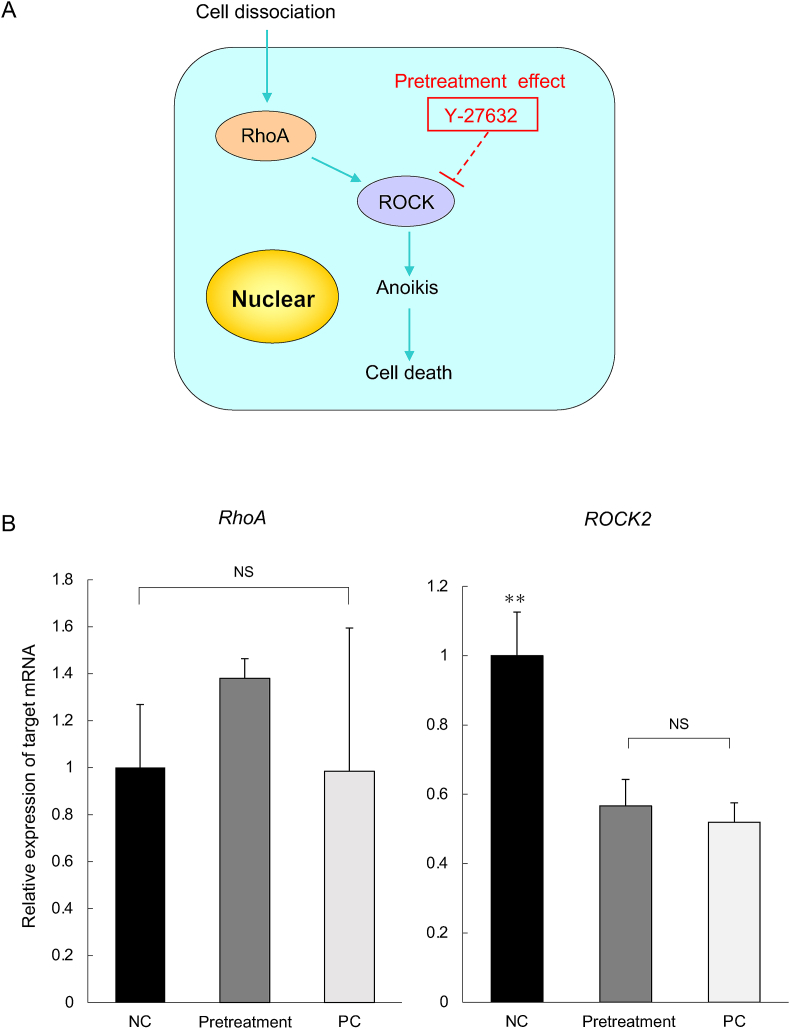
mRNA expression analysis of RPE cells after transient exposure of suspension culture to Y-27632. (A) Schematic of relationship between Y-27632 and cell death. RPE cell dissociation triggers apoptotic mechanisms via RhoA/ROCK signaling pathway that progress to cell death and Y-27632 involvement. (B) mRNA expression level of *RhoA* and *ROCK2* in human RPE cells pretreated with 10 μM Y-27632 after 4 h suspension culture (ΔΔCt to *β-actin*). Data are shown as mean ± SED (n = 4). One-way ANOVA with Tukey's post hoc pairwise comparison tests were performed. NC, negative control; PC, positive control.

To clarify Y-27632 function, we evaluated the expression level of RhoA and ROCK2 in human RPE cells after transient exposure to 10 μM Y-27632 and incubation of suspension culture for 4 h. Cell dissociation induces cell apoptosis via the activation of the RhoA/ROCK signaling pathway, in which RhoA is upstream of ROCK and ROCK1 and ROCK2 are serine/threonine kinases that are downstream targets of RhoA (Fig. 4A) [21]. A significant difference in the relative expressions of RhoA mRNA was not seen (Fig. 4B). Next, the expression of ROCK2 was significantly downregulated upon transient exposure to Y-27632. Transient exposure to Y-27632 demonstrated a clear inhibitory effect on ROCK even after 4 h.

## 4 Discussion

Although a variety of beneficial effects of ROCK inhibitors were reported in a number of research fields [[22], [23], [24], [25], [26]], there are no studies demonstrating the persistence of its effects after drug removal. In this study, we demonstrated that the beneficial effects of Y-27632 persist after its removal from the culture medium, which may be clinically advantageous as host tissues would not need to be exposed to ROCK inhibitors, e.g., in the case of transplantation.

One of the most important benefits of ROCK inhibitors that has been demonstrated in lung epithelial cells is its antiapoptotic effect by inhibiting disruption of the actin cytoskeleton [27]. In addition, we demonstrated that it was particularly effective in protecting RPE cell suspensions at body temperature, which usually evokes anoikis [4]. This study confirmed the cellular protective effect of transient exposure to high concentrations of Y-27632 (10, 30, and 100 μM) (Fig. 2A), which was better than NC (without ROCK inhibitor treatment) at all time points (4 and 8 h) (Fig. 2B). However, this study has some limitations, i.e., the need for higher Y-27632 concentration at pretreatment and shorter continuation time compared with the PC, probably due to the gradual decrease in Y-27632 concentration in each single RPE cell under its effective concentration by their molecular metabolism [28] and/or molecular diffusion to the medium [29]. Application of the effective conditions found in this study in practical settings will provide huge advantages in maintaining cell viability during storage at single cell suspension before their application.

As a functional characterization of ROCK inhibitor-pretreated RPE cells, cell proliferation was initially validated by recovery culture after transient exposure, which can be expected as a cell source of the regenerate RPE cell layer for its transplantation [3]. Improvement in cell proliferation by transient exposure was observed at days 1 and 2 after recovery culture and was not seen after day 3 because it was believed that the cells were so confluent that it was difficult to interpret any difference (Fig. 3A). After transient exposure and culturing for 10 days, it was confirmed that transient exposure to Y-27632 improved the number of viable cells compared with NC, which is in line with a previously reported study that also used Y-27632 in the medium [30]. In addition, Y-27632 did not affect cell morphology in any group in this study (Fig. 3B), suggesting that the actin filaments and other cytokeratins that typically decline on activation of the ROCK signaling pathway were not damaged [27]. In addition, VEGF secretion was also not affected. These results suggest that preservation had no effect on the VEGF secretion capacity of the surviving RPE cells. Thus, the RPE cells may retain the ability to secrete important proteins even after being preserved.

For further analysis on the ROCK signaling pathway, mRNA was checked using qPCR to confirm the mechanism of action of Y-27632. ROCK2 is an isoform of the ROCK family of kinases [[31], [32], [33]] and highly expressed in the eye [5]. We confirmed that ROCK2 was downregulated, similar to the PC and pretreatment after 4 h suspension culture (Fig. 4B). However, we did not confirm a difference in the expression level of RhoA, which is upstream of ROCK in the Rho/ROCK signaling pathway (Fig. 4B). Y-27632 is an inhibitor that specifically targets ROCK in the Rho/ROCK signaling pathway [34], and we showed that ROCK2 was inhibited even when Y-27632 was removed from the medium. Our data demonstrates that Y-27632 acts on the Rho/ROCK signaling pathway with only transient exposure, i.e., its presence in the medium is not required.

## 5 Conclusion

In conclusion, this study demonstrated that the effect of Y-27632 sustained at various conditions even after removal from the medium. The Y-27632 concentration demonstrated in this study was the one with high versatility in clinical practice. We shed light on the important effects of Y-27632 persistence so as to decrease the side effects. The fact that the effectiveness was shown while avoiding the risk of side effects meant that it has the potential for application in various types of novel medical treatments.

## Author contributions

S.K. and Y.T. conceived and designed the experiments. H.I. performed all experiments and S.K. and H.I wrote the manuscript, which was approved by all authors prior to submission. Y.T., T.I., and K.K. provided helpful guidance and suggestions. Y.T. and K.K. directed all experimental work.

## Declaration of competing interest

The authors have no conflicts of interest directly relevant to the content of this article.
